# CCR5+ CD8+ T Cells Are Associated with Poor Response to PD-1 Blockade Therapy

**DOI:** 10.3390/ijms27114963

**Published:** 2026-05-30

**Authors:** Ziheng Zhao, Yuwei Liu, Zhaofei Wu, Chunliang Qi, Yingze Ning, Yiting Lin, Xuewen Pang, Guangliang Qiang, Wei Wang

**Affiliations:** 1 NHC Key Laboratory of Medical Immunology, Department of Immunology, School of Basic Medical Sciences, Peking University, 38 Xueyuan Road, Haidian District, Beijing 100191, China; zhaoziheng@pku.edu.cn (Z.Z.); liuyuwei0416@163.com (Y.L.); wuzhaofei@pku.edu.cn (Z.W.); 1810305202@pku.edu.cn (C.Q.); 2011110013@pku.edu.cn (Y.L.); pangxuewen@bjmu.edu.cn (X.P.); 2 Department of Thoracic Surgery, Peking University Third Hospital, 49 Huayuan North Road, Haidian District, Beijing 100191, China; ningyingze@163.com

**Keywords:** CCR5, PD-1 blockade therapy, CD8+ T cells, exhaustion

## Abstract

Many patients develop poor clinical response to immune checkpoint inhibitors (ICIs), especially PD-1/PD-L1 blockade. However, transcriptomic features of chemokine receptors associated with poor response remain incompletely characterized. We analyzed publicly available single-cell RNA sequencing datasets from non-small-cell lung cancer (NSCLC) and melanoma cohorts, with additional exploratory analyses in hepatocellular carcinoma (HCC) and colorectal cancer datasets. Chemokine receptor expression on CD8+ T cells from clinical responsive and non-responsive samples to anti-PD-1 therapy was systematically profiled. Differential gene expression, cell-state scoring, pseudotime trajectory inference, and ligand–receptor interaction analysis were performed to characterize associated transcriptional states and predicted cellular interactions. *CCR5* transcript expression in tumor-infiltrating CD8+ T cells was associated with lower responsiveness to PD-1/PD-L1 blockade therapy. CCR5+ CD8+ T cells exhibited transcriptional features associated with increased exhaustion, reduced stemness, and advanced differentiation. Pseudotime inference suggested progressively increased *CCR5* expression along the inferred differentiation trajectory. Ligand–receptor interaction analysis further identified predicted interactions between CCR5+ CD8+ T cells and tumor-associated myeloid cells, with elevated expression of *CCL3* and *CCL4* observed in myeloid populations from non-responsive tumors. Together, these findings identify transcriptomic associations between CCR5+ CD8+ T cell states and poor clinical response to PD-1 blockade therapy. These observations support the CCL3/4–CCR5 axis as a candidate pathway for future spatial, functional, and experimental validation.

## 1. Introduction

The immune checkpoint inhibitors (ICIs), such as antibodies targeting programmed cell death protein-1 (PD-1) and its ligand (PD-L1), have yielded clinical benefits for patients with advanced malignancies and have become a standard of care for numerous cancers [1,2]. By blocking inhibitory signaling pathways, ICIs reinvigorate the anti-tumor functions of tumor-infiltrating T cells [3,4]. Nevertheless, only a subset of patients responds to ICI therapy, and many who initially benefit eventually develop acquired resistance, substantially limiting the clinical reach of ICIs [5,6]. A deep understanding of the underlying mechanisms of poor response to ICIs is therefore critical for developing effective strategies to sensitize clinical response.

The functional state of T cells, particularly their differentiation and exhaustion status within the tumor microenvironment (TME), is a fundamental determinant of ICI efficacy [7,8,9,10]. It is now clear that tumor-infiltrating CD8+ T cells are not a homogeneous population but rather exist along a continuous spectrum of differentiation states, ranging from self-renewing “progenitor exhausted” (Tpex) cells to terminally differentiated “terminally exhausted” (Tex) cells that have lost proliferative capacity and effector function [7,9,10]. Tpex cells, often marked by the transcription factor TCF1, are considered the primary population that responds to PD-1 blockade and mediates tumor clearance, whereas the accumulation of Tex cells is strongly associated with therapeutic poor response [8,10]. Elucidating the molecular drivers that push T cells toward a terminally exhausted fate is therefore paramount for understanding and sensitizing ICI response.

The chemokine and chemokine receptor system is a critical regulatory network that orchestrates the trafficking, positioning, and interaction of immune cells, thereby shaping the cellular composition and architecture of the TME and profoundly influencing anti-tumor immunity [11,12,13]. Different chemokine axes can recruit distinct subsets of immune cells. For instance, the CXCL9/10/11-CXCR3 axis is generally associated with the recruitment of effector T cells and favorable clinical outcomes [11,12,13]. Conversely, other axes, such as the CCL2-CCR2 axis, primarily recruit immunosuppressive myeloid cells, fostering an immunosuppressive TME [11,12,13]. We hypothesized that a specific chemokine receptor might exist that uniquely marks and is associated with the accumulation or retention of dysfunctional, terminally exhausted T cells within the TME, thus directly contributing to PD-1 blockade poor response. However, such a chemokine receptor directly linking T cell terminal exhaustion to ICI response has remained elusive.

In this study, we performed a systematic integrative analysis of public multi-cohort single-cell transcriptomic datasets, primarily from NSCLC and melanoma, to identify chemokine receptors associated with poor clinical response to PD-1 blockade. We identified CCR5 as a transcriptomic marker consistently associated with non-responsive CD8+ T cell states, including features linked to advanced differentiation and reduced stemness. Ligand–receptor interaction analysis further suggested predicted interactions between CCR5+ CD8+ T cells and ligand-expressing myeloid populations. Increased expression of *CCL3* and *CCL4* in myeloid cells from non-responsive tumors was associated with transcriptional programs linked to T cell exhaustion. Together, these findings provide transcriptomic insights into cellular states associated with poor ICI response and support the CCL3/4–CCR5 axis as a candidate pathway for future functional and spatial validation.

## 2. Results

### 2.1. CCR5+ CD8+ T Cells Are Significantly Enriched in Tumors from Patients with Poor Response to PD-1 Blockade

**Figure 1:**
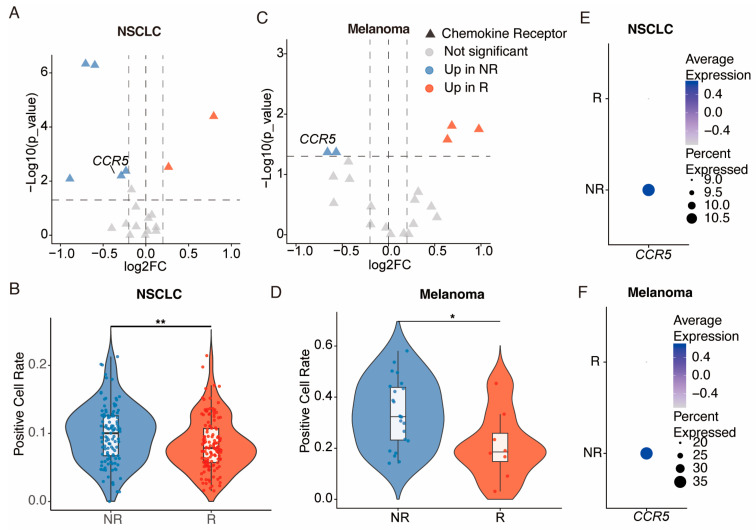
The proportion of CCR5+ CD8+ T cells is significantly increased in patients resistant to anti-PD-1 therapy. (**A**) Volcano plot showing the differential proportion of chemokine receptor-positive cells in CD8+ T cells from the non-small-cell lung cancer (NSCLC) cohort, comparing clinical responsive (R) (N = 130) versus non-responsive (NR) (N = 112) samples. (**B**) Violin plots with superimposed boxplots comparing the percentage of CCR5+ cells within the CD8+ T cell compartment in the NSCLC cohort. Each dot represents an individual patient sample. (**C**) Volcano plot as in (**A**) but for the melanoma cohort with R (N = 8) and NR (N = 23) samples. (**D**) Violin plots as in (**B**) but for the melanoma cohort. (**E**,**F**) Dot plots showing the expression of *CCR5* in CD8+ T cells from clinical responsive and non-responsive samples in (**E**) NSCLC and (**F**) melanoma cohorts. Statistical significance was determined using a Wilcoxon rank-sum test. * *p* < 0.05, ** *p* < 0.01.

**Figure 2:**
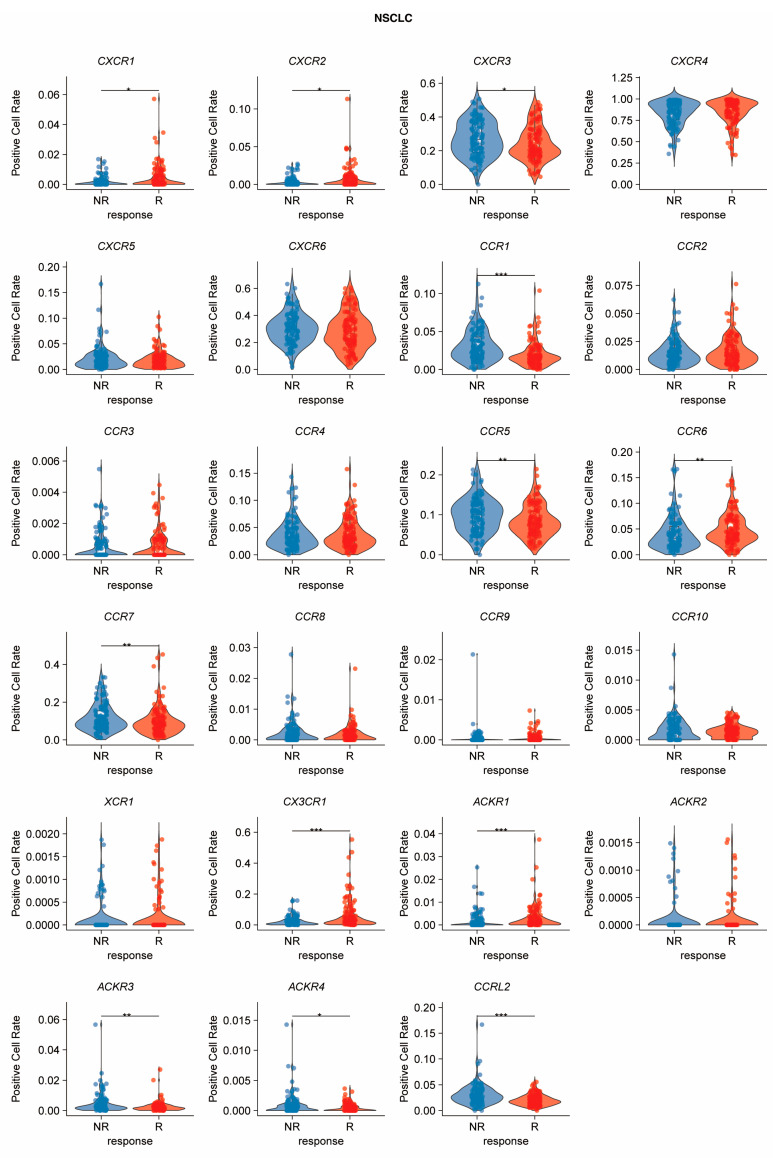
Proportion of chemokine receptor-positive CD8+ T cells in the NSCLC cohort. Violin plots with superimposed boxplots comparing the percentage of cells positive for each indicated chemokine receptor within the CD8+ T cell compartment between clinical responsive (N = 130) and non-responsive (NR) (N = 112) samples. Statistical significance was determined using a Wilcoxon rank-sum test. * *p* < 0.05, ** *p* < 0.01, *** *p* < 0.001.

**Figure 3:**
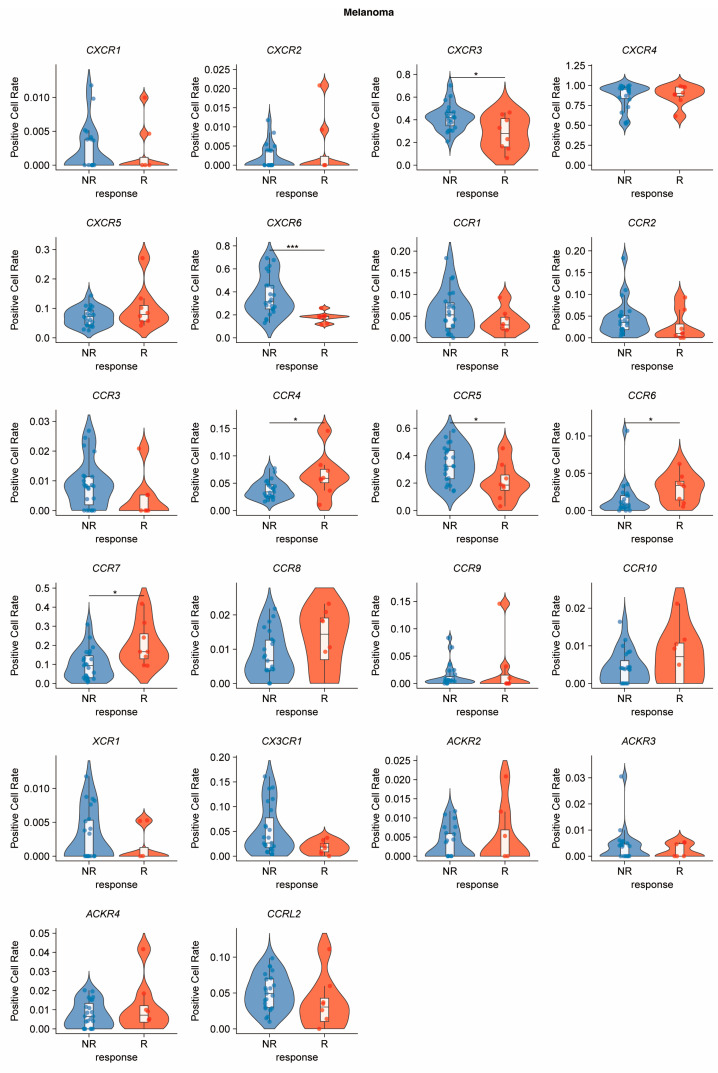
Proportion of chemokine receptor-positive CD8+ T cells in the melanoma cohort. Violin plots with superimposed boxplots comparing the percentage of cells positive for each indicated chemokine receptor within the CD8+ T cell compartment between clinical responsive (R) (N = 8) and non-responsive (NR) (N = 23). Each dot represents an individual sample. Statistical significance was determined using a Wilcoxon rank-sum test. * *p* < 0.05, *** *p* < 0.001.

To identify chemokine receptors associated with poor clinical response to anti-PD-1 therapy, we first analyzed public single-cell RNA sequencing (scRNA-seq) datasets from patients with non-small-cell lung cancer [14] (NSCLC; GSE243013) and melanoma [15] (GSE120575) (Table S1). First, we annotated the cells with global immune cell types based on the original study annotations and auto-annotation tools. The annotations were confirmed by the expression of well-established feature genes (Figure S1A,B). Notably, CD8+ T cells expressing canonical markers (*CD3D*, *CD8A*, and *CD8B*) were selected for downstream analysis. We defined receptor-positive cells as those with log-normalized gene expression greater than 0.5 based on a sensitivity test (Table S3). By comparing clinical responsive (R) and non-responsive (NR) tumor samples to PD-1 blockade, we observed a consistent and significant increase in the proportion of CCR5+ cells within the CD8+ T cells in NR samples across both cancer types (Figure 1A–D). In contrast, other chemokine receptors did not show a consistent pattern of differential enrichment between R and NR samples (Figure 2 and Figure 3, Tables S4 and S5). A similar trend was observed in exploratory HCC and CRC datasets [16,17] (Figure S1C,D), which is consistent with the potential association between elevated *CCR5* expression, increased frequency of CCR5+ CD8+ T cells, and poor response to immunotherapy.

**Figure 4:**
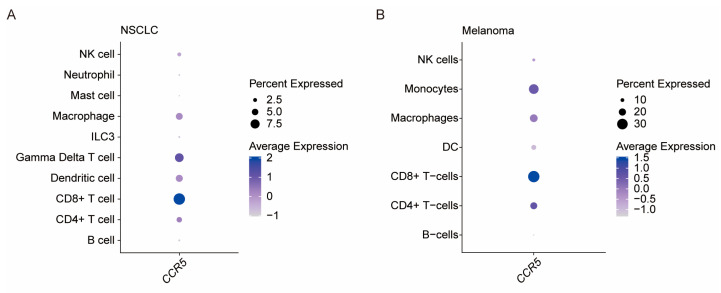
*CCR5* expression across tumor-infiltrating immune cell populations. (**A**,**B**) Dot plot showing the expression level and percentage of *CCR5* expressing cells across all major tumor-infiltrating immune cell populations in (**A**) NSCLC and (**B**) melanoma.

To understand the expression landscape of *CCR5* within the tumor microenvironment (TME), we analyzed its distribution across various tumor-infiltrating immune cell types (Figure 4). In both NSCLC and melanoma, *CCR5* was expressed across multiple immune lineages but was particularly enriched and highly expressed in CD8+ T cells (Figure 4).

Collectively, these results suggest that the increased frequency of CCR5+ CD8+ T cells is associated with poor clinical response to PD-1 blockade.

### 2.2. CCR5+ CD8+ T Cells Exhibit a Transcriptional Profile of Advanced Differentiation and Low Stemness

**Figure 5:**
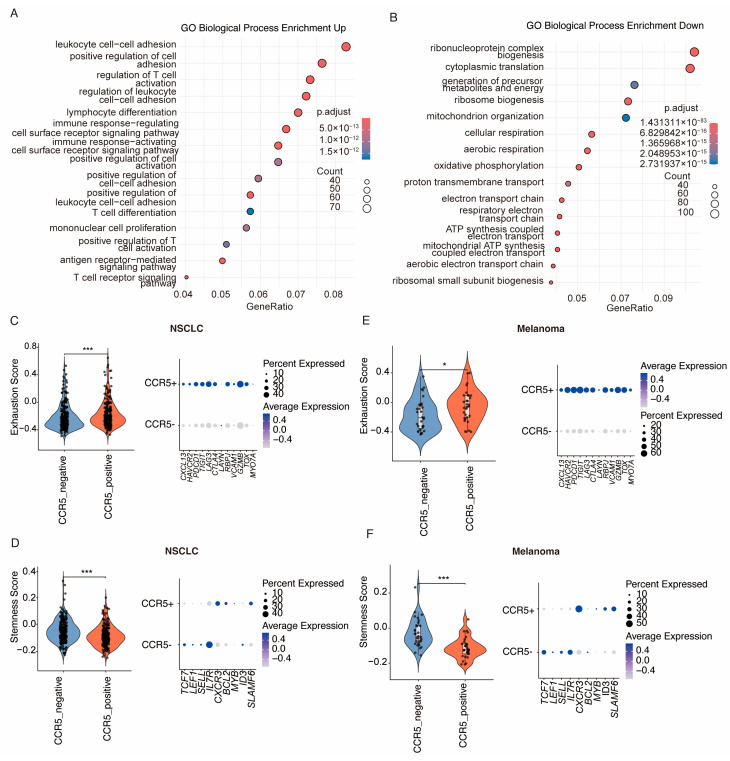
CCR5+ CD8+ T cells exhibit increased exhaustion and decreased stemness signatures compared to CCR5− cells. (**A**,**B**) Gene Ontology (GO) enrichment analysis for biological processes (BPs) of genes significantly (**A**) upregulated and (**B**) downregulated in CCR5+ versus CCR5− CD8+ T cells. (**C**) Left: Violin plots comparing exhaustion scores between CCR5+ and CCR5− CD8+ T cells at the sample level in the NSCLC cohort (N = 234). Right: Dot plot showing the expression of key exhaustion-associated genes. (**D**) Left: Violin plots comparing stemness scores between CCR5+ and CCR5− CD8+ T cells at the sample level in the NSCLC cohort (N = 234). Right: Dot plot showing the expression of key stemness-associated genes. (**E**,**F**) Same analyses as in (**C**,**D**) but for the melanoma cohort (N = 24). Statistical significance was determined using a Wilcoxon rank-sum test. * *p* < 0.05, *** *p* < 0.001.

To investigate the molecular features associated with CCR5+ CD8+ T cells in the context of poor clinical response to immunotherapy, we performed pseudobulk differential gene expression (DGE) analysis between CCR5+ and CCR5− CD8+ T cells from the NSCLC cohort. Compared to CCR5−, 1032 genes were significantly upregulated and 1096 genes were significantly downregulated in CCR5+ CD8+ T cells (Table S6). Gene Ontology (GO) enrichment analysis revealed that upregulated genes were primarily enriched in Gene Ontology biological processes related to cell chemotaxis, T cell activation and T cell differentiation (Figure 5A and Table S7). Intriguingly, downregulated genes were significantly associated with metabolic and biosynthetic processes (Figure 5B and Table S8).

T cell exhaustion has been associated with immunotherapy failure. Based on our initial findings that CCR5+ T cells upregulated genes associated with the T cell activation process (Figure 5A), we calculated exhaustion and stemness scores for CCR5+ and CCR5− CD8+ T cells at the sample level (Table S9). Consistent with their enrichment in NR samples, CCR5+ T cells displayed significantly higher exhaustion scores (Figure 5C) and markedly lower stemness scores (Figure 5D). This trend was also observed in the melanoma cohort (Figure 5E,F). Specifically, key markers of terminal differentiation/exhaustion phenotype, including *CXCL13*, *HAVCR2* (TIM-3), *PDCD1* (PD-1), *TIGIT*, *LAG3*, *CTLA4*, *TOX*, and *GZMB*, were consistently upregulated in CCR5+ T cells. Conversely, while a few stemness-associated genes linked to activation (e.g., *MYB*) were elevated, genes critical for maintaining stemness—such as the precursor exhausted T cell markers *TCF7* (TCF1) and *LEF1*, and the memory T cell markers *IL7R* and *SELL* (CD62L)—were significantly downregulated in CCR5+ T cells (Figure 5C,D).

To verify the robustness of our observation, we tested multiple independent gene signatures, including one exhaustion signature excluding *GZMB* and *CXCL13*, and independent stemness signatures with modified gene compositions (e.g., replacing *CXCR3* with *CXCR5* and *CCR7*) (Table S9). Across different gene sets, CCR5+ subsets consistently exhibited increased exhaustion scores (Figure S2A,B) while also exhibiting decreased stemness scores (Figure S2C,D).

Together, these results suggest that CCR5+ CD8+ T cells exhibit transcriptional characteristics of a highly differentiated subpopulation associated with functional exhaustion and a loss of self-renewal potential.

### 2.3. CCR5 Expression Correlates with the Differentiation Trajectory of CD8+ T Cells Toward Exhaustion

**Figure 6:**
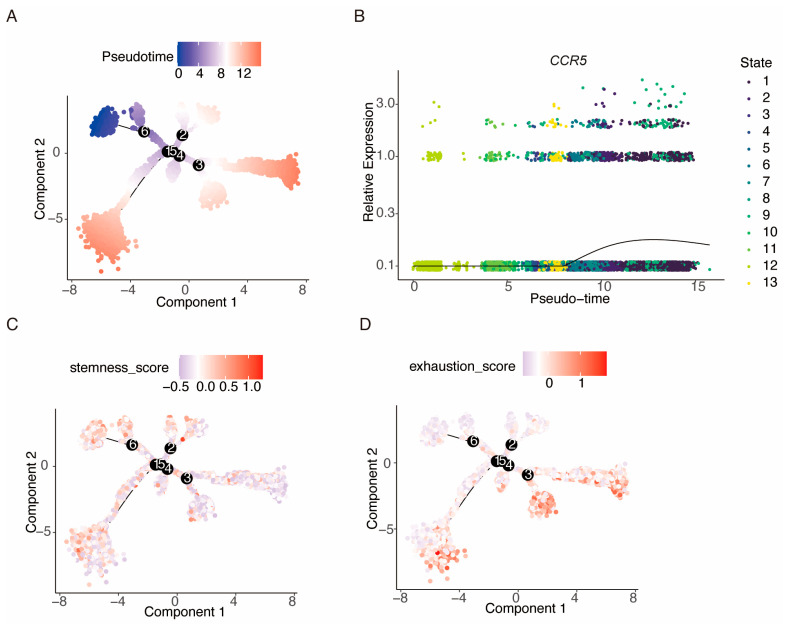
*CCR5* expression increases along the T cell differentiation trajectory. (**A**) CD8+ T cells colored by pseudotime, with the root of the trajectory indicating a more stem-like state. (**B**) Expression of CCR5 plotted along the pseudotime trajectory for CD8+ T cells from the NSCLC cohort. (**C**,**D**) Stemness scores (**C**) and exhaustion scores (**D**) plotted along the pseudotime trajectory, showing opposing trends during differentiation. The numbered black circles represent key branch nodes, where cells transition into distinct differentiation lineages.

To further investigate the relationship between *CCR5* expression and T cell differentiation state, we performed pseudotime analysis on CD8+ T cells from the NSCLC cohort to computationally reconstruct a potential differentiation trajectory (Figure 6A). This analysis ordered cells along a continuous transcriptomic path spanning from a stem-like to a terminally exhausted phenotype. While we recognize that pseudotime inference derived from cross-sectional single-cell data represents a static computational modeling of cellular states rather than a true chronological or causal mechanism, we observed a progressive increase in *CCR5* expression as CD8+ T cells advanced along the pseudotime trajectory (Figure 6B). Concomitantly, the stemness score gradually decreased (Figure 6C), while the exhaustion score steadily increased (Figure 6D). These results suggest a consistent transcriptomic association between CCR5 expression and the progressive shift toward terminal differentiation and exhaustion. These findings remain correlative and reflect inferred cellular state transitions rather than direct developmental trajectories, warranting future prospective functional studies to determine definitive developmental causality.

### 2.4. CCR5 Is Preferentially Expressed in Highly Differentiated CD8+ T Cell Subsets That Accumulate in Non-Responding Patients

**Figure 7:**
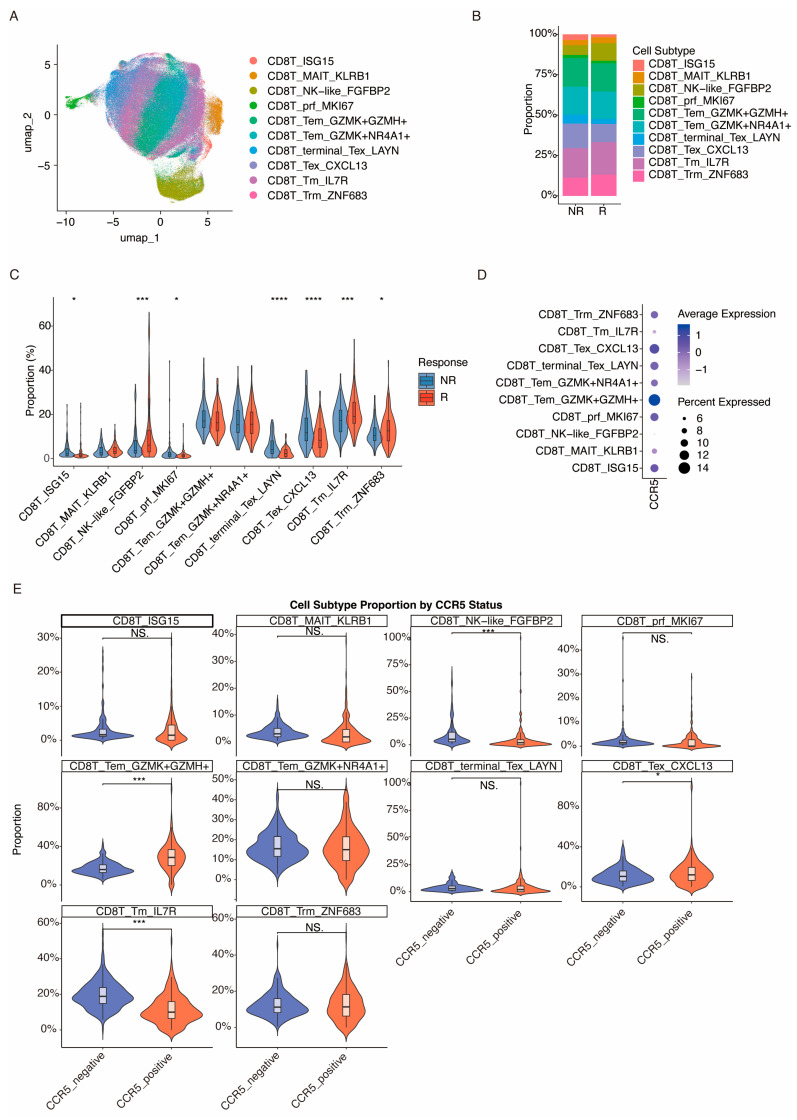
CCR5 is preferentially expressed in highly differentiated CD8+ T cell subsets. All analyses were performed in R (N = 130) and NR (N = 112) NSCLC patients. (**A**) UMAP plot showing the identified subclusters of CD8+ T cells. (**B**) Stacked bar plot showing the proportion of each CD8+ T cell subset in R and NR. (**C**) Violin plots comparing the proportion of each major CD8+ T cell subset. (**D**) Dot plot showing the expression level and percentage of CCR5−expressing cells across all identified CD8+ T cell subclusters. (**E**) Violin plots showing the cellular composition of the CCR5+ and CCR5− CD8+ T cell pools, highlighting the enrichment of specific subsets. Statistical significance was determined using Wilcoxon rank-sum test. NS, not significant; * *p* < 0.05, *** *p* < 0.001, **** *p* < 0.0001.

To characterize the cellular identity of CCR5−expressing cells, we performed fine-grained clustering of CD8+ T cells from the NSCLC cohort, resolving 10 distinct subsets based on published annotations [14] (Figure 7A). Specifically, memory-like features were captured in CD8T_Tm_IL7R (IL7R), while tissue-resident characteristics were defined by CD8T_Trm_ZNF683 (*ZNF683*, *XCL1/2*) (Figure S3A and Table S10). The exhausted T cell lineages, CD8T_Tex_CXCL13 and CD8T_terminal_Tex_LAYN, were clearly demarcated by the robust upregulation of *CXCL13*, *LAYN*, and immune checkpoints (*CTLA4*, *HAVCR2*) (Figure S3A and Table S10). Effector memory heterogeneity was reflected by two GZMK+ subsets, while CD8T_NK-like_FGFBP2 and CD8T_ISG15 clusters demonstrated distinct cytotoxic (*FGFBP2*, *CX3CR1*) and interferon-stimulated (*ISG15*, *IFIT3*) signatures, respectively (Figure S3A and Table S10). Lastly, proliferation-specific markers (*MKI67*, *TYMS*) exclusively isolated the cycling population into the CD8T_prf_MKI67 cluster (Figure S3A and Table S10). Together, these distinct cluster-specific expression profiles support the robustness of the CD8+ T cell clustering stategy.

First, comparing subset proportions between groups, we found that tumors from NR samples were significantly enriched in terminally exhausted subsets (CD8T_Tex_CXCL13, CD8T_terminal_Tex_LAYN) but depleted of the stem-like resident memory subset CD8T_Trm_IL7R (Figure 7B,C), corroborating our earlier findings.

Next, we assessed the expression pattern of CCR5 across these subsets. Consistent with the pseudotime analysis, CCR5 was most highly expressed in activated and terminally differentiated subsets, including effector memory (CD8T_Tem_GZMK+GZMH+) and the two exhausted populations (CD8T_Tex_CXCL13, CD8T_terminal_Tex_LAYN), while its expression was low in the more quiescent, stem-like memory subset CD8T_Trm_IL7R (Figure 7D).

Finally, we directly compared the cellular composition of the CCR5+ and CCR5− CD8+ T cell pools. This analysis indicated that, relative to the CCR5− pool, the CCR5+ pool was significantly enriched for the effector memory (CD8T_Tem_GZMK+GZMH+) and exhausted (CD8T_Tex_CXCL13) subsets, while being dramatically depleted of the stem-like CD8T_Trm_IL7R subset (Figure 7E).

To further examine the association between CCR5 expression and functional states across CD8+ T cell subsets, we evaluated exhaustion and stemness scores at the sample level within each subset. Across most subsets, CCR5 expression was associated with lower stemness scores and higher exhaustion scores, including CD8T_ISG15, CD8T_prf_MKI67, CD8T_Tem_GZMK+NR4A1+, and CD8T_Tm_IL7R (Figure S3B,C, Table S11). This pattern suggests that CCR5 expression is preferentially observed in CD8+ T cell states characterized by reduced stemness and increased exhaustion.

Taken together, our analysis outlines a compositional shift within the CCR5+ population, which appears to be preferentially composed of terminally differentiated effector and exhausted T cell states rather than stem-like T cells.

### 2.5. CCR5+ CD8+ T Cells Show Increased Predicted Ligand–Receptor Communication with Tumor-Associated Myeloid Cells

**Figure 8:**
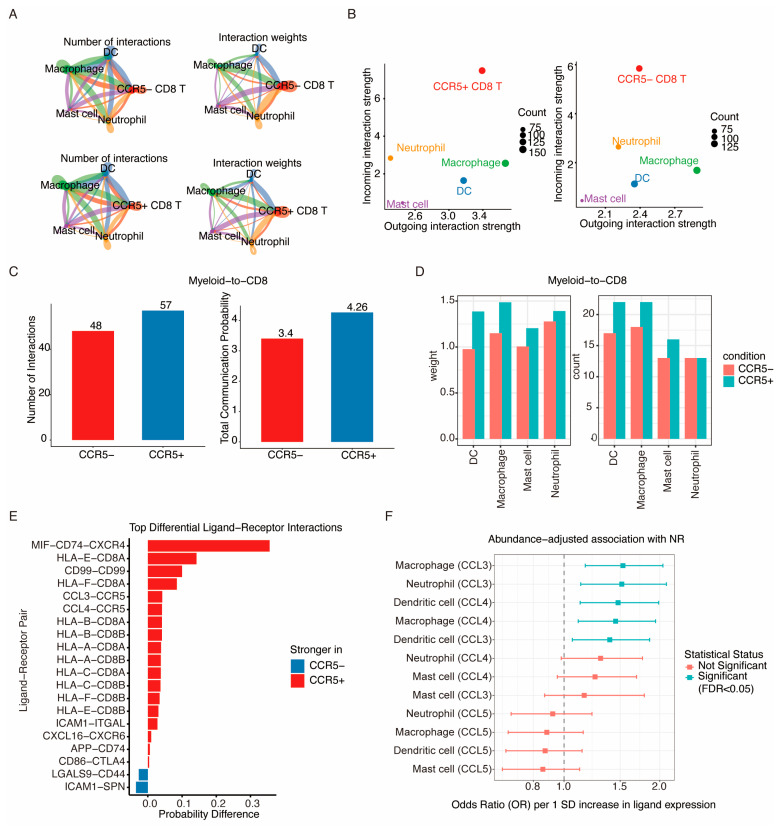
CCR5+ CD8+ T cells show increased predicted interactions with myeloid cells. (**A**) Chord diagrams illustrating CellChat-inferred ligand–receptor interaction number and communication weight between CCR5+ or CCR5− CD8+ T cells and major myeloid cell populations (dendritic cells [DC], macrophages, mast cells, and neutrophils) in the NSCLC TME. (**B**) Comparison of inferred network centrality scores for interactions between CCR5−defined CD8+ T cells and myeloid populations. (**C**) Bar plots comparing the overall number of predicted interactions (left) and total inferred communication probability (right) from all myeloid cells to CCR5+ versus CCR5− CD8+ T cells. (**D**) Bar plots showing the same comparison as in (**C**) for each individual myeloid cell subset. (**E**) Bar plots showing the top differential predicted ligand–receptor interactions from myeloid cells targeting CCR5+ versus CCR5− CD8+ T cells. (**F**) Abundance adjusted logistic reg for myeloid cell derived CCR5 ligands.

Given the highly differentiated and exhausted phenotype of CCR5+ CD8+ T cells, we hypothesized that their transcriptional state may be associated with signals from other immune populations within the TME. We therefore performed ligand–receptor-based cell–cell communication inference using CellChat at the transcriptional level, focusing on the crosstalk between CD8+ T cells and myeloid cells, including macrophages, dendritic cells, mast cells and neutrophils. The analysis revealed a higher number and greater total inferred communication probability of predicted interactions between CCR5+ CD8+ T cells and myeloid subsets compared to their CCR5− counterparts (Figure 8A–D and Table S12). These results suggest that CCR5+ CD8+ T cells are associated with an increased inferred ligand–receptor communication network with tumor-associated myeloid cells.

Delving into the specific signaling pathways, we found that beyond the expected chemokine signaling, several HLA–CD8-related predicted ligand–receptor interactions were enriched between myeloid cells and CCR5+ T cells (Figure 8E and Table S12). Because these interactions were inferred from dissociated single-cell transcriptomic data, they should not be interpreted as direct evidence of persistent antigen presentation, antigen specificity, or TCR stimulation. Rather, these findings indicate that CCR5+ CD8+ T cells may be embedded in a transcriptional communication network containing antigen-presentation-related ligand–receptor signals. This observation is hypothesis-generating and requires further validation by complementary approaches such as HLA antigen-presentation machinery analysis, TCR clonotype tracing, spatial transcriptomics, or multiplex immunostaining.

Furthermore, we examined the expression of the CCR5 ligands—*CCL3*, *CCL4*, and *CCL5*. These chemokines were broadly expressed by tumor-associated myeloid cells and lymphocytes (Figure S4A). To dissect the cellular sources contributing to these differences, we compared the expression of these ligands in every immune subset at sample level. Notably, *CCL3* expression was significantly upregulated in non-responders across multiple immune subsets, showing prominent statistical significance in macrophages, CD4 + T cells, CD8+ T cells, neutrophils, B cells, and dendritic cells (Figure S4B, Tables S13 and S14). Similarly, *CCL4* levels were significantly elevated in non-responders within DCs, macrophages, and neutrophils, while demonstrating a slight but significant increase in responders within NK cells (Figure S4B, Tables S13 and S14). In contrast, *CCL5* expression remained largely unaltered across most cell types between the two groups, with the sole exception of a significant upregulation in responders within the CD4 + T cell compartment (Figure S4B, Tables S13 and S14). Together, these findings indicate that elevated *CCL3* and *CCL4* expression across multiple immune compartments is associated with a non-responsive phenotype, highlighting their potential relevance to treatment outcome.

To determine whether CCR5 ligands serve as independent predictors of non-response, an abundance-adjusted logistic regression analysis was performed (Figure 8F, Table S15). After controlling for cell type proportions, a one standard deviation (SD) increase in the expression of specific myeloid-derived ligands was significantly associated with a higher likelihood of being a non-responder (NR, Odds Ratio > 1.0, FDR < 0.05). Notably, macrophage-derived *CCL3* and neutrophil-derived *CCL3* exhibited the strongest risk correlations, both achieving an OR of approximately 1.5. Significant positive associations with non-response were also observed for dendritic cell-derived *CCL4*, macrophage-derived *CCL4*, and dendritic cell-derived *CCL3* (all FDR < 0.05). Conversely, while certain ligands derived from neutrophils, mast cells, and macrophages showed trends toward altered risk, their confidence intervals crossed the reference line (OR = 1.0), and they did not meet the threshold for statistical significance (FDR ≥ 0.05). These data demonstrate that elevated expression of *CCL3* and *CCL4* by specific myeloid compartments—independent of overall cell abundance—is associated with a non-responsive clinical phenotype.

To further elucidate the relationship between chemokine production and cellular infiltration, we correlated the weighted mean ligand expression in myeloid cells with the total myeloid cell abundance per patient for *CCL3*, *CCL4*, and *CCL5* (Figure S4C). Spearman correlation analysis revealed a highly significant positive correlation between myeloid cell abundance and *CCL3* expression (r = 0.32, *p* = 7.68 × 10^−7^), as well as a weaker but statistically significant positive correlation with CCL4 expression (r = 0.15, *p* = 0.02). In contrast, *CCL5* expression displayed a weak but significant negative correlation with total myeloid cell abundance (r = −0.16, *p* = 0.01). These findings indicate that myeloid infiltration is associated with increased per-cell transcriptional expression of *CCL3* and *CCL4*, suggesting coordinated regulation of chemokine expression and immune infiltration within the tumor microenvironment.

### 2.6. Evaluation of CCR5 as a Potential Biomarker Associated with PD-1 Blockade Responsiveness

**Figure 9:**
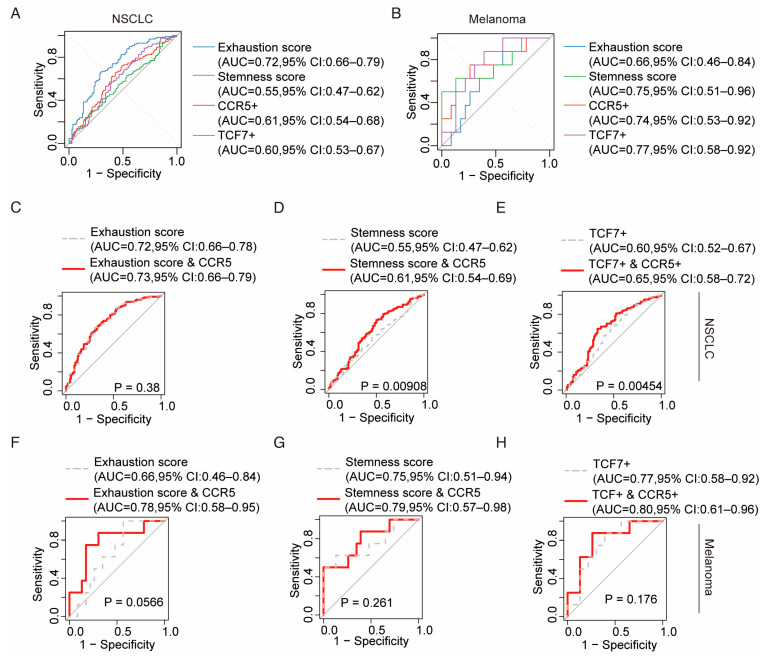
Evaluation of CCR5 as a potential biomarker associated with PD-1 blockade responsiveness. (**A**,**B**) ROC curves evaluating predictors. ROC analysis of exhaustion score, stemness score, CCR5 positive rate, and TCF1 positive rate in the NSCLC cohort (**A**) and melanoma cohort (**B**). The area under the curve (AUC) and 95% confidence interval (95% CI) for each biomarker is indicated in the legend. (**C**–**E**) Additive contribution of CCR5 in the NSCLC cohort. ROC curves comparing established signatures alone (gray dashed lines) with their respective combinations with CCR5 positive rate (red solid lines). The combinations include (**C**) exhaustion score, (**D**) stemness score and (**E**) TCF1 positive rate. (**F**–**H**) Synergistic contribution of CCR5 in the melanoma cohort. ROC curves assessing the predictive gain of adding CCR5 to (**F**) exhaustion score, (**G**) stemness score or (**H**) TCF7. Statistical analysis was performed using a likelihood ratio test (LRT) test to compare AUCs between single and combined models. *p* values were marked and considered significant when <0.05.

While T cell stemness, exhaustion states, and TCF1+ (encoded by *TCF7*) populations are established predictors of PD-1 blockade responsiveness [8,14], we sought to investigate the discriminative performance of CCR5 and its relationship with these existing signatures via receiver operating characteristic (ROC) analysis (Table S16). In the NSCLC cohort, exhaustion score showed the strongest standalone discrimination with an AUC at 0.72 (95% CI: 0.66–0.79) (Figure 9A); whereas, CCR5 exhibited only modest predictive performance with an AUC at 0.61 (95% CI: 0.54–0.68), similar to stemness score (AUC: 0.55, 95% CI: 0.47–0.62) and TCF7-positive fraction (AUC: 0.60, 95% CI: 0.53–0.67) (Figure 9A). In the melanoma cohort, CCR5 again showed limited discriminative ability relative to the established signatures (AUC: 0.74, 95% CI: 0.53–0.92) (Figure 9B).

To assess the incremental predictive value of the CCR5+ rate, we compared nested logistic regression models with and without CCR5 using a likelihood ratio testing. In NSCLC, adding CCR5 did not materially improve the predictive performance of the exhaustion model (Figure 9C) and yielded only small or statistically limited gains when added to stemness (*p* = 0.00908)- or *TCF7* (*p* = 0.00454)-based models (Figure 9D,E), with delta AUC at 0.06 and 0.05 separately. Furthermore, in the melanoma cohort, the addition of CCR5 did not yield any further enhancement in predictive power beyond existing factors (Figure 9F–H). Collectively, these results suggest that CCR5 has limited standalone discriminative ability and minimal incremental value in the presence of stronger established transcriptional response signatures.

## 3. Discussion

Through an in-depth, multi-cohort analysis of public single-cell sequencing datasets, our study characterizes the chemokine receptor CCR5 as a transcriptomic feature associated with advanced differentiation and reduced stemness of CD8+ T cells in tumors with poor clinical response to PD-1 blockade. Our central finding is that CCR5+ CD8+ T cells are significantly enriched in tumors that are non-responsive to anti-PD-1 therapy and exhibit transcriptional programs associated with a state of advanced differentiation and low stemness. These findings identify CCR5 expression as a marker associated with dysfunctional CD8+ T cell states rather than establishing a direct mechanistic role in resistance. Conceptually, we propose a model wherein myeloid cells within the non-responsive tumor microenvironment upregulate the CCR5 ligands CCL3 and CCL4. Ligand–receptor interaction analysis suggested that this axis may be associated with predicted interactions between CCR5+ T cells and myeloid populations, potentially tracking with reduced stemness and poor response to PD-1 blockade therapy.

The role of CCR5 in immunity is complex and context dependent. As a receptor for several pro-inflammatory chemokines (CCL3, CCL4, CCL5), CCR5 is integral to normal immune responses, mediating the migration of T cells, macrophages, and other leukocytes to sites of inflammation [18,19,20]. In some cancer studies, *CCR5* expression has been associated with increased T cell infiltration and favorable prognosis [20,21]. In contrast, our transcriptomic analyses suggest that within the chronically stimulated tumor microenvironment, *CCR5* expression is associated with CD8+ T cell states characterized by advanced differentiation and reduced stemness. This observation is consistent with recent discoveries in chronic viral infection and cancer, where persistent TCR and inflammatory signaling induces the upregulation of various chemokine receptors on exhausted T cells [22,23]. Our findings therefore support a transcriptomic association between elevated *CCR5* expression and more differentiated CD8+ T cell states in tumors with poor response to PD-1 blockade.

The predicted interactions between CCR5+ T cells and myeloid populations identified in our study provide a possible framework for future mechanistic investigation. However, these ligand–receptor interaction analyses are computational inferences and do not establish direct antigen presentation, physical proximity, antigen specificity, or persistent TCR stimulation. Therefore, these observations should be interpreted as hypothesis-generating transcriptomic associations rather than evidence of a defined biological mechanism. Future studies integrating spatial transcriptomics, multiplex imaging, TCR lineage tracing, or functional perturbation approaches will be required to validate these proposed cellular interactions.

Our findings suggest that the CCL3/4–CCR5 axis may represent a candidate pathway associated with T cell dysfunction and poor immunotherapy response. While CCR5 antagonists like Maraviroc are safe anti-HIV drugs [24,25] currently being explored in oncology [26,27,28], the therapeutic relevance of CCR5 blockade in this context remains speculative. Future experimental studies will be required to determine whether CCR5 inhibition can modulate T cell states or improve responsiveness to PD-1 blockade therapy.

This study has several limitations. First, all conclusions are derived from retrospective analyses of public scRNA-seq datasets and therefore remain correlative in nature. Second, functional validation experiments, including perturbation of CCR5 signaling in vitro or in vivo, were not performed. Third, although pseudotime and ligand–receptor analyses suggested potential relationships between *CCR5* expression, T cell differentiation states, and myeloid interactions, these approaches cannot establish true temporal progression or causal cellular communication. Finally, scRNA-seq analysis loses the spatial context of the tissue. Therefore, direct spatial proximity between CCR5+ T cells and ligand-expressing myeloid cells could not be confirmed. Future spatial transcriptomic and multiplex imaging studies will be necessary to validate these predicted interactions within the tumor microenvironment.

## 4. Materials and Methods

### 4.1. Software Environment

All analyses were performed in the R (version 4.5.1; R Foundation for Statistical Computing, Vienna, Austria) statistical environment. Single-cell RNA sequencing analyses were primarily conducted using the Seurat package (version 5.4.0; Satija Lab, New York, NY, USA), including data normalization, dimensional reduction, clustering, differential expression analysis, and module score calculation.

Data manipulation and preprocessing were performed using packages from the tidyverse ecosystem (Hadley Wickham & contributors, http://tidyverse.org/), including dplyr (version 1.1.4), tidyr (version 1.3.2), tibble (version 3.3.0), purr (version 1.2.2), and stringr (version 1.6.0). Sparse matrix operations were handled using the Matrix (version 1.7-4; R Core Team, Vienna, Austria) package.

Visualization was performed using ggplot2 (version 4.0.1; Hadley Wickham, Houston, TX, USA) together with auxiliary packages including ggpubr (version 0.6.3; Alboukadel Kassambara, http://rpkgs.datanovia.com/ggpubr/, accessed on 25 May 2026), ggrepel (version 0.9.6; Kamil Slowikowski, http://github.com/slowkow/ggrepel, accessed on 25 May 2026), patchwork (version 1.3.2; Thomas Lin Pedersen, http://github.com/thomasp85/patchwork, accessed on 25 May 2026), pheatmap (version 1.0.13; Raivo Kolde, http://github.com/raivokolde/pheatmap, accessed on 25 May 2026), scales (version 1.4.0; Hadley Wickham, Houston, TX, USA), forcats (version 1.0.1; Hadley Wickham, Houston, TX, USA), and ggsignif (version 0.6.4; Constantin Ahlmann-Eltze, http://github.com/const-ae/ggsignif, accessed on 25 May 2026).

Functional enrichment analyses were conducted using clusterProfiler (version 4.16.0; Southern Medical University, Guangzhou, China) in combination with the human gene annotation database org.Hs.eg.db (version 3.21.0). Gene identifier conversion was performed using clusterProfiler-compatible annotation functions.

Pseudobulk differential expression analyses were performed using the edgeR (version 4.6.3) and limma (version 3.64.3) workflows, including voom transformation and empirical Bayes moderation.

Predictive modeling and statistical analyses were conducted using pROC (version 1.19.0.1) for receiver operating characteristic (ROC) analysis, lmtest (version 0.9-40) for likelihood ratio testing, and broom (version 1.0.13) for model statistic extraction and tabulation.

Optional trajectory inference analyses were performed using monocle (version 2.36.0). Optional cell–cell communication analyses were conducted using CellChat (version 2.1.2) with the CellChatDB.human ligand–receptor interaction database.

### 4.2. Single-Cell RNA Sequencing Data Acquisition and Preprocessing

Single-cell RNA sequencing (scRNA-seq) data were obtained from the Gene Expression Omnibus (GEO) database under the accession number GSE243013 (NSCLC) [14], GSE120575 (melanoma) [15], GSE206325 (HCC) [17] and GSE205506 (colon cancer) [16]. All the datasets information could be found in Table S1. For all subsequent analyses, Seurat objects were constructed using the preprocessed expression matrices or .rds files and metadata provided with each dataset. The data were normalized using the LogNormalize method and scaled for downstream analyses of the immune cell compartment. Patient clinical information was used to classify samples into clinical responsive (R) and non-responsive (NR) groups.

### 4.3. Cell Type Annotation

The cell type annotation for the NSCLC cohort is based on the fine-grained sub_cell_type annotations provided in the dataset; we grouped cells into broader immune lineages using keyword matching (e.g., “CD8T_”, “Mφ_”). These major categories included CD8+ T cells, CD4+ T cells, B cells, macrophages, dendritic cells, and NK cells, which were used for high-level analyses of gene expression and cellular composition. As for the melanoma, HCC and CRC cohorts, all the cells were annotated by SingleR (version 2.10.0) package with the BlueprintEncodeData database. The major annotation was confirmed by well-established markers.

### 4.4. Screening of Chemokine Receptor Expression Profiles

To systematically screen for chemokine receptors associated with response to PD-1 blockade, we initiated our analysis within the CD8+ T cell population. For each chemokine receptor gene, cells with a log-normalized expression > 0.5 were defined as receptor-positive, which is stable among all the thresholds at 0.0, 0.2, 0.5, and 0.8 (Table S2). We then calculated the proportion of these positive cells within the total CD8+ T cell population for each patient sample. The Wilcoxon rank-sum test was used to compare these proportions between the R and NR sample groups. The significance threshold was set at an adjusted *p*-value < 0.05 and an absolute log2 fold change (log2FC) > 0.2. Results were visualized using volcano plots via ggplot2 (version 4.0.1).

### 4.5. In-Depth Analysis of the CD8+ T Cell Compartment

CD8+ T cells were subsetted for independent downstream analysis. Using the standard Seurat (version 5.4.0) workflow, highly variable genes were identified using the FindVariableFeatures function, followed by principal component analysis (PCA). Uniform Manifold Approximation and Projection (UMAP) was used for visualization following clustering. To identify marker genes defining each CD8+ T cell subcluster, we employed the FindAllMarkers function, which performs a Wilcoxon rank-sum test. Markers were filtered based on expression in at least 25% of cells within a cluster and a log2FC > 0.25.

### 4.6. Cellular Composition Analysis

To evaluate differences in cell composition between clinical response groups or between different CCR5 expression states, we calculated the relative abundance (proportion) of each cell subset on a per-sample basis. Specifically, the number of cells in each subset was divided by the total number of cells in the corresponding sample. Differences in proportions between groups were visualized using stacked bar plots and statistically assessed using a Wilcoxon rank-sum test.

### 4.7. Differential Gene Expression (DGE) Analysis

To investigate the transcriptional signatures of CCR5+ CD8+ T cells, we stratified CD8+ T cells into CCR5+ (CCR5_positive; Log-normalized expression greater than 0.5) and CCR5− (CCR5_negative) groups. DGE analysis was performed using the FindMarkers function in Seurat (version 5.4.0), implementing a Wilcoxon rank-sum test. Genes with a Benjamini–Hochberg adjusted *p*-value (*p*_val_adj) < 0.05 and an absolute log2FC > 0.1 were considered significantly differentially expressed.

Gene Ontology (GO) enrichment analysis for the biological process (BP) category was performed on the differentially expressed genes using the R package clusterProfiler (version 4.16.0). Gene symbols were first converted to Entrez IDs using the org.Hs.eg.db (version 3.21.0) annotation database. Analyses were conducted separately for upregulated and downregulated gene sets. GO terms with a Benjamini–Hochberg adjusted *p*-value < 0.05 were considered significantly enriched.

### 4.8. Gene Module Score Analysis

To quantify the activity of specific biological processes, gene module scores were calculated using the AddModuleScore function in Seurat (version 5.4.0). We utilized curated gene lists from the published literature corresponding to T cell exhaustion and T cell stemness. These scores were used to compare the functional states of CCR5+ and CCR5− CD8+ T cells. Differences between groups were visualized using violin plots, with statistical significance assessed using the ggsignif (version 0.6.4) package. The gene lists used are as follows: Exhaustion genes [29,30]: *CXCL13, HAVCR2, PDCD1, TIGIT, LAG3, CTLA4, LAYN, RBPJ, VCAM1, GZMB, TOX, MYO7A*; Stemness genes [8,31]: *TCF7*, *LEF1*, *SELL*, *IL7R*, *CXCR3*, *BCL2*, *MYB* [32], *ID3*, *SLAMF6*.

### 4.9. Cell–Cell Communication Analysis

Cell–cell communication was inferred using CellChat (version 2.1.2) from log-normalized RNA expression matrices extracted from Seurat objects. Separate CellChat analyses were performed for the CCR5+ and CCR5− CD8 T cell groups by merging each CD8 subset with the myeloid compartment. Broad cell types were annotated as DC, macrophage, neutrophil, mast cell, and CCR5+ or CCR5− CD8 T cells. The human ligand–receptor reference database CellChatDB.human (integrated in CellChat version 2.1.2) was used. For each object, the standard CellChat workflow was applied, including subsetData, identifyOverExpressedGenes, identifyOverExpressedInteractions, computeCommunProb, filterCommunication (min.cells = 10), and computeCommunProbPathway. Interactions supported by fewer than 10 cells were excluded. Myeloid-to-CD8 interactions were extracted using subsetCommunication, and the two CellChat objects were merged for comparative pathway-level analysis. Because no explicit batch correction or cell-abundance matching was performed, inferred communication strengths should be interpreted cautiously as relative ligand–receptor interaction probabilities rather than direct evidence of physical cell contact, persistent antigen presentation, or TCR stimulation.

### 4.10. Predictive Modeling of Immunotherapy Response

For predictive modeling, single-cell transcriptomic data from CD8+ T cells were aggregated at the patient level. For each sample, the fraction of CCR5+ and TCF7+ CD8+ T cells was calculated using a predefined normalized-expression threshold, and exhaustion and stemness scores were summarized as patient-level mean values. Clinical response was encoded as a binary variable (response = 1, non-response = 0).

The predictive performance of CCR5 and established transcriptional markers was evaluated using ROC analysis with the pROC package (version 1.19.0.1). Standalone ROC curves were generated for CCR5+ fraction, exhaustion score, stemness score, and TCF7-positive fraction. To estimate the incremental contribution of CCR5 beyond established markers, nested logistic regression models were fitted and compared using a likelihood ratio test (LRT) with the lmtest package (version 0.9-40). Specifically, models including exhaustion, stemness, or TCF7 were compared against corresponding models with additional CCR5+ fraction. AUC values were reported as descriptive measures of discrimination, and model comparison was based on LRT rather than AUC alone. Correlations between CCR5+ fraction and other response-associated signatures were assessed using Spearman’s rank correlation.

Logistic regression analyses included both univariable and adjusted multivariable models. Continuous predictors were standardized prior to modeling such that odds ratios represented the effect per one standard deviation increase.

Adjusted models incorporated baseline covariates when available, including CD8+ T cell abundance, dataset identity, major immune subset abundance, and myeloid subtype abundance.

For each logistic regression model, regression coefficients, standard errors, Wald statistics, odds ratios, 95% confidence intervals, *p*-values, and false discovery rates were calculated using the broom package (version 1.0.13).

Correlations between CCR5+ fractions and other response-associated features were assessed using Spearman rank correlation.

### 4.11. Trajectory Analysis

To model the dynamic differentiation continuum of CD8+ T cells, unsupervised pseudotime trajectory analysis was performed using the Monocle 2 R package (version 2.26.0). Individual cells were downsampled to a maximum threshold of 10,000 cells to optimize computational efficiency. A CellDataSet object was initialized using the raw count matrix, and size factors and expression dispersions were estimated under a negative binomial empirical dispersion model. Trajectory-defining ordering features were restricted to the top 2000 highly variable genes (HVGs) identified in the Seurat (version 5.4.0) workflow using the setOrderingFilter function. Manifold learning and dimensional reduction were executed via the Discriminative Dimensionality Reduction with Trees (DDRTree) algorithm, and cells were subsequently ordered along the learned principal graph using the orderCells function.

To establish developmental directionality, the trajectory’s root branch was dynamically defined as the specific state containing the highest stemness score calculated as described above. Expression kinetics of CCR5 and module scores along the continuous pseudotime axis were modeled using local regression smoothing.

### 4.12. Statistical Analysis and Visualization

All data processing, statistical analyses, and visualizations were performed in the R (version 4.5.1) programming environment. Key packages included Seurat (version 5.4.0) for scRNA-seq analysis, dplyr (version 1.1.4) and tidyr (version 1.3.2) for data manipulation, ggplot2 (version 4.0.1), ggpubr (version 0.6.3), and patchwork (version 1.3.2) for visualization, and clusterProfiler (version 4.16.0) for functional enrichment analysis. Unless otherwise specified, comparisons between two groups were performed using a Wilcoxon rank-sum test. A *p*-value < 0.05 was considered statistically significant.

## 5. Conclusions

Our study suggests that *CCR5* transcript detection represents a potential marker of CD8+ T cell terminal exhaustion that is associated with poor response to PD-1 blockade. Based on transcriptomic profiling, we characterize a potential regulatory axis whereby crosstalk between myeloid cells and T cells via the CCL3/4-CCR5 axis is associated with T cell dysfunction, though further protein-level validation is required to definitively confirm secreted chemokine availability and functional receptor engagement. These findings deepen our understanding of ICI response and provide bioinformatic insights into the development of novel combination immunotherapies targeting CCR5, which warrants further experimental and functional validation.

## Data Availability

The data that support the findings of this study are openly available in the Gene Expression Omnibus (GEO) database under accession numbers GSE243013 (https://www.ncbi.nlm.nih.gov/geo/query/acc.cgi?acc=GSE243013, accessed on 13 August 2025), GSE120575 (https://www.ncbi.nlm.nih.gov/geo/query/acc.cgi?acc=GSE120575, accessed on 24 September 2025), GSE206325 (https://www.ncbi.nlm.nih.gov/geo/query/acc.cgi?acc=GSE206325, accessed on 24 September 2025) and GSE205506 (https://www.ncbi.nlm.nih.gov/geo/query/acc.cgi?acc=GSE205506, accessed on 24 September 2025). The codes generated in this study are available in github (https://github.com/zhaoziheng97-ux/Rcode-for-CCR5-CD8-T-Cells-are-Associated-with-Poor-Response-to-PD-1-Blockade-Therapy/blob/main/script.R, accessed on 24 September 2025).

## Supplementary Materials

The following supporting information can be downloaded at: https://www.mdpi.com/article/10.3390/ijms27114963/s1. Reference [33] is cited in the supplementary materials.

## Abbreviations

The following abbreviations are used in this manuscript:

AUC	Area under the curve
CCL	C-C Motif Chemokine Ligand
CCR	C-C Motif Chemokine Receptor
CRC	Colorectal Cancer
CXCL	C-X-C Motif Chemokine Ligand
CXCR	C-X-C Motif Chemokine Receptor
DC	Dendritic Cell
DGE	Differential Gene Expression
FC	Fold Change
GEO	Gene Expression Omnibus
GO	Gene Ontology
GZMB	Granzyme B
HCC	Hepatocellular Carcinoma
HLA	Human Leukocyte Antigen
ICI	Immune Checkpoint Inhibitor
MDSC	Myeloid-Derived Suppressor Cell
NK cell	Natural Killer cell
NR	Non-responsive
NSCLC	Non-Small-Cell Lung Cancer
PCA	Principal Component Analysis
PD-1	Programmed Cell Death Protein-1
PD-L1	Programmed Cell Death Ligand-1
R	Responsive
ROC	Receiver operating characteristic
scRNA-seq	Single-cell RNA sequencing
TCR	T Cell Receptor
Tem	Effector Memory T cell
Tex	Terminally Exhausted T cell
TME	Tumor Microenvironment
Tpex	Progenitor Exhausted T cell
Treg	Regulatory T cell
Trm	Resident Memory T cell
UMAP	Uniform Manifold Approximation and Projection
