# Supplementary material for: CCR5+ CD8+ T Cells Are Associated with Poor Response to PD-1 Blockade Therapy
Source: Int J Mol Sci. 2026 May 30;27(11):4963. doi: 10.3390/ijms27114963 (PMC13256934; doi:10.3390/ijms27114963)
Supplement: Supplementary file 1 [file ijms-27-04963-s001.zip › Supplementary figures.docx]

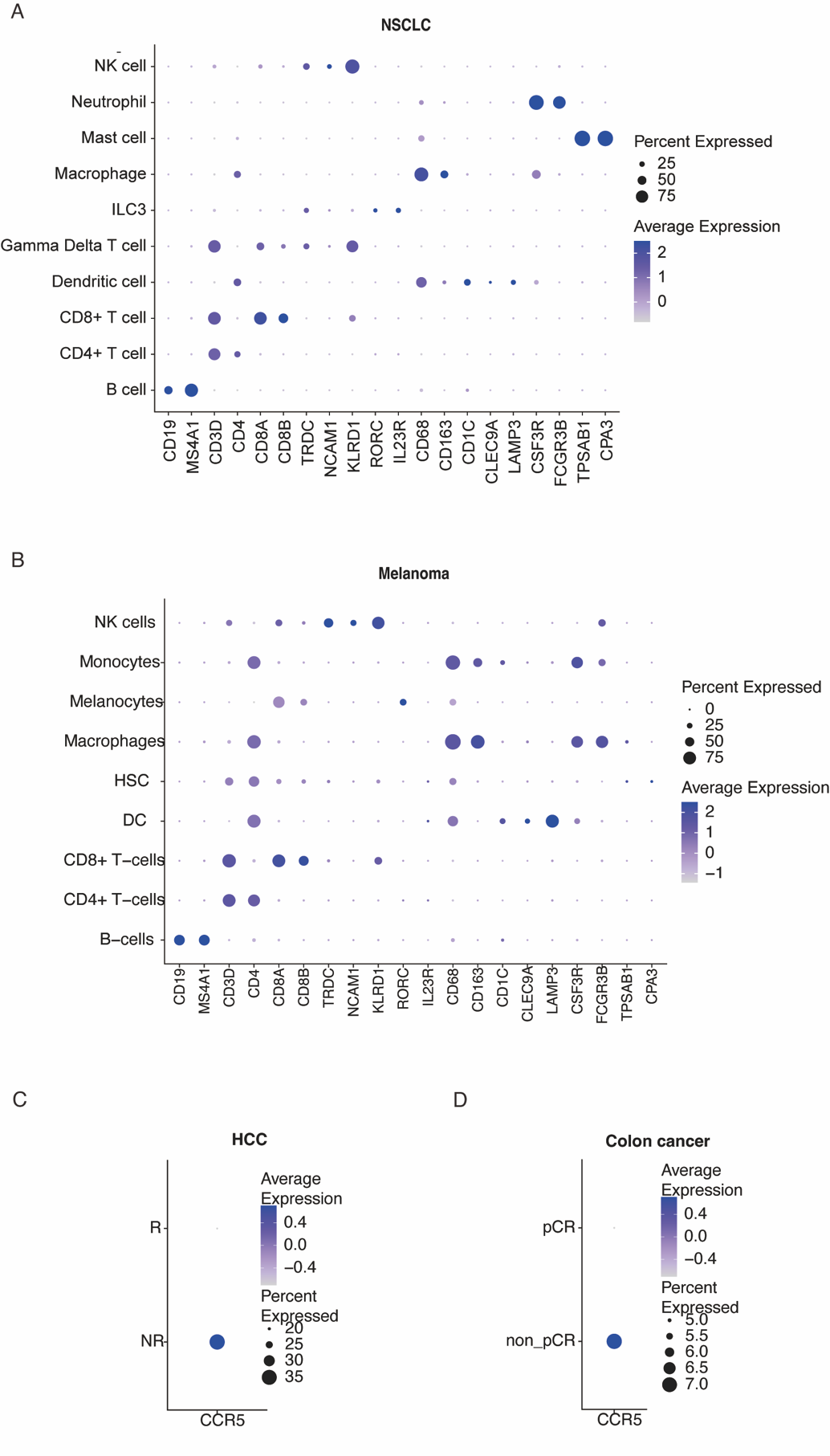


**Supplementary figure1. Confirmation of annotation and CCR5 expression in HCC and CRC derived CD8+ T cells.** (A and B) Dotplot of well-established feature genes of cells from NSCLC(A) and melanoma cohorts(B). (C and D) Dot plots showing the expression of CCR5 in CD8+ T cells from responsive and less-responsive samples from hepatocellular carcinoma (C), and (D) colon cancer (CRC) cohorts.

**
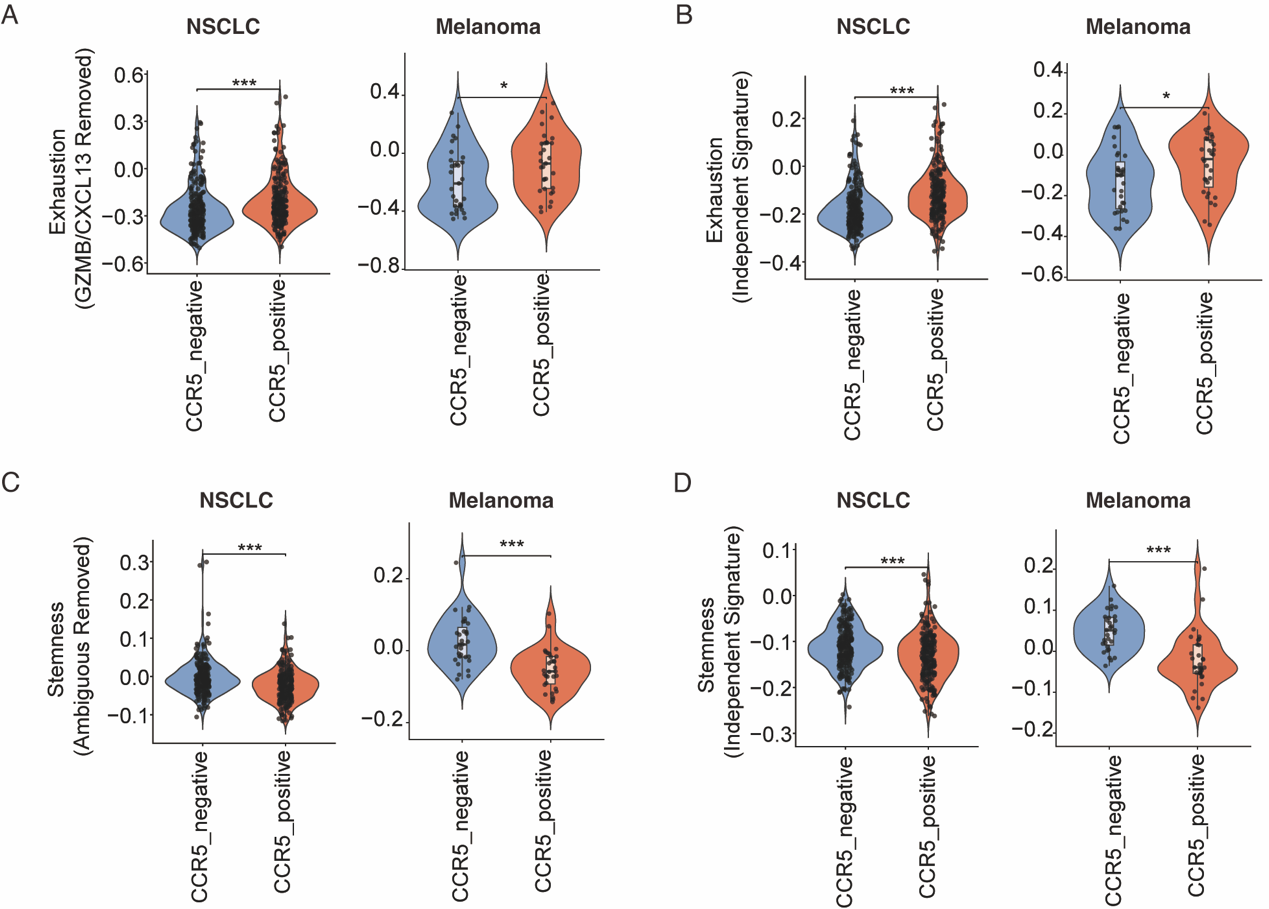
**

**Supplementary figure2. Association of CCR5 status with T-cell exhaustion and stemness signatures in NSCLC and melanoma.** (A–D) Violin plots illustrating the module scores for T-cell exhaustion and stemness across CCR5-negative (blue) and CCR5-positive (orange) groups in NSCLC(N=234) and melanoma(N=24) cohorts.(A) Exhaustion scores calculated after removing GZMB and CXCL13 expression.(B) Exhaustion scores derived from an independent gene signature.(C) Stemness scores calculated after removing ambiguous gene signatures.(D) Stemness scores derived from an independent gene signature. Statistical significance was assessed using the Wilcoxon rank-sum test; NS, not significant; * P < 0.05, **P < 0.01, *** P < 0.001.

**
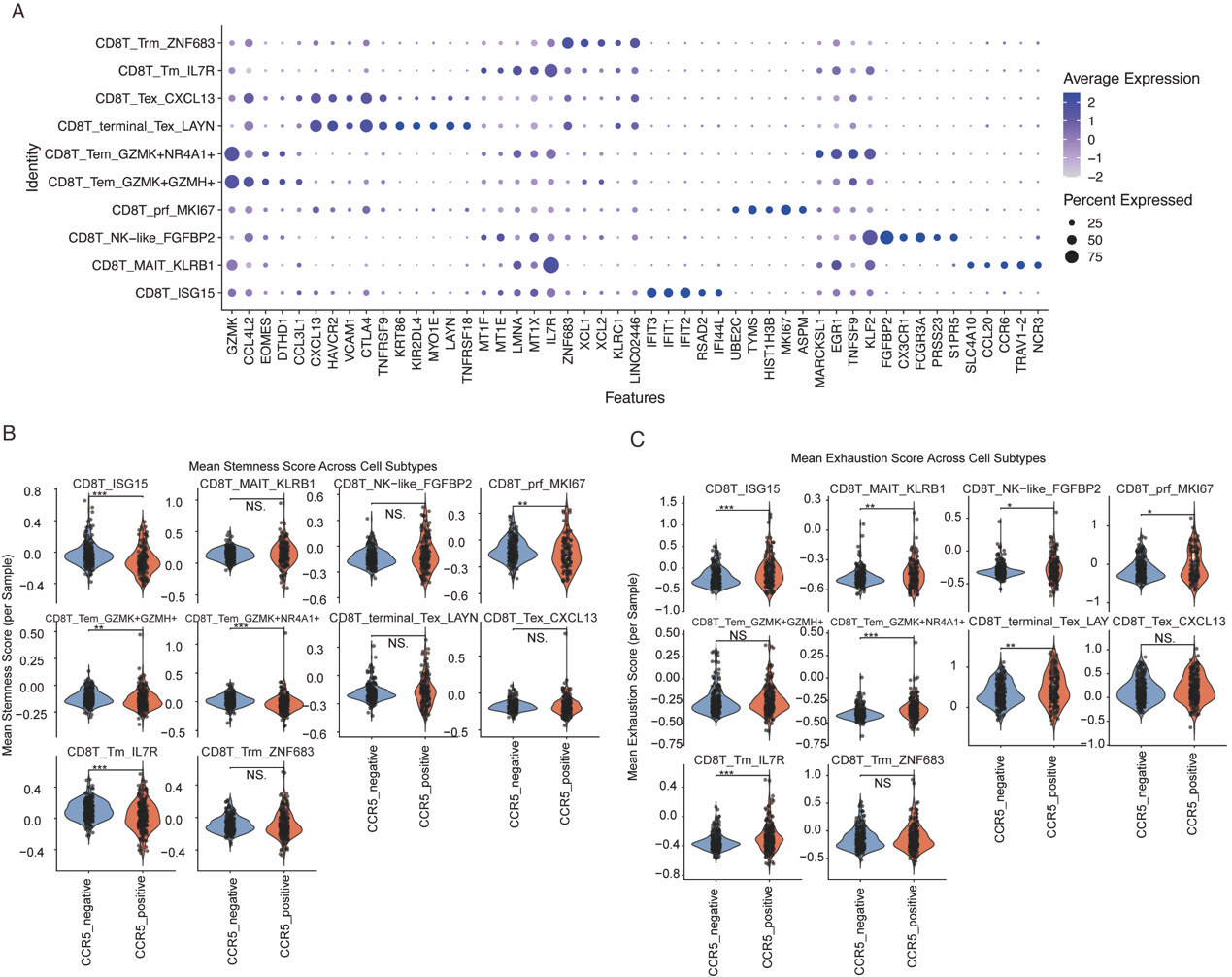
**

**Figure S3. Marker gene expression and signature scores across CD8+ T-cell subsets.**

(A) Dot plot showing the expression of selected marker genes across ten annotated CD8+ T-cell clusters. (B and C) Violin plots comparing the mean stemness scores (B) and mean exhaustion scores (C) between CCR5-negative (blue) and CCR5-positive (orange) groups across the ten CD8+ T-cell subtypes from NSCLC cohort(N=234). Each data point represents an individual patient sample (aggregated at the sample level).Statistical significance was assessed using the Wilcoxon rank-sum test; NS, not significant; * P < 0.05, **P < 0.01, *** P < 0.001.

**
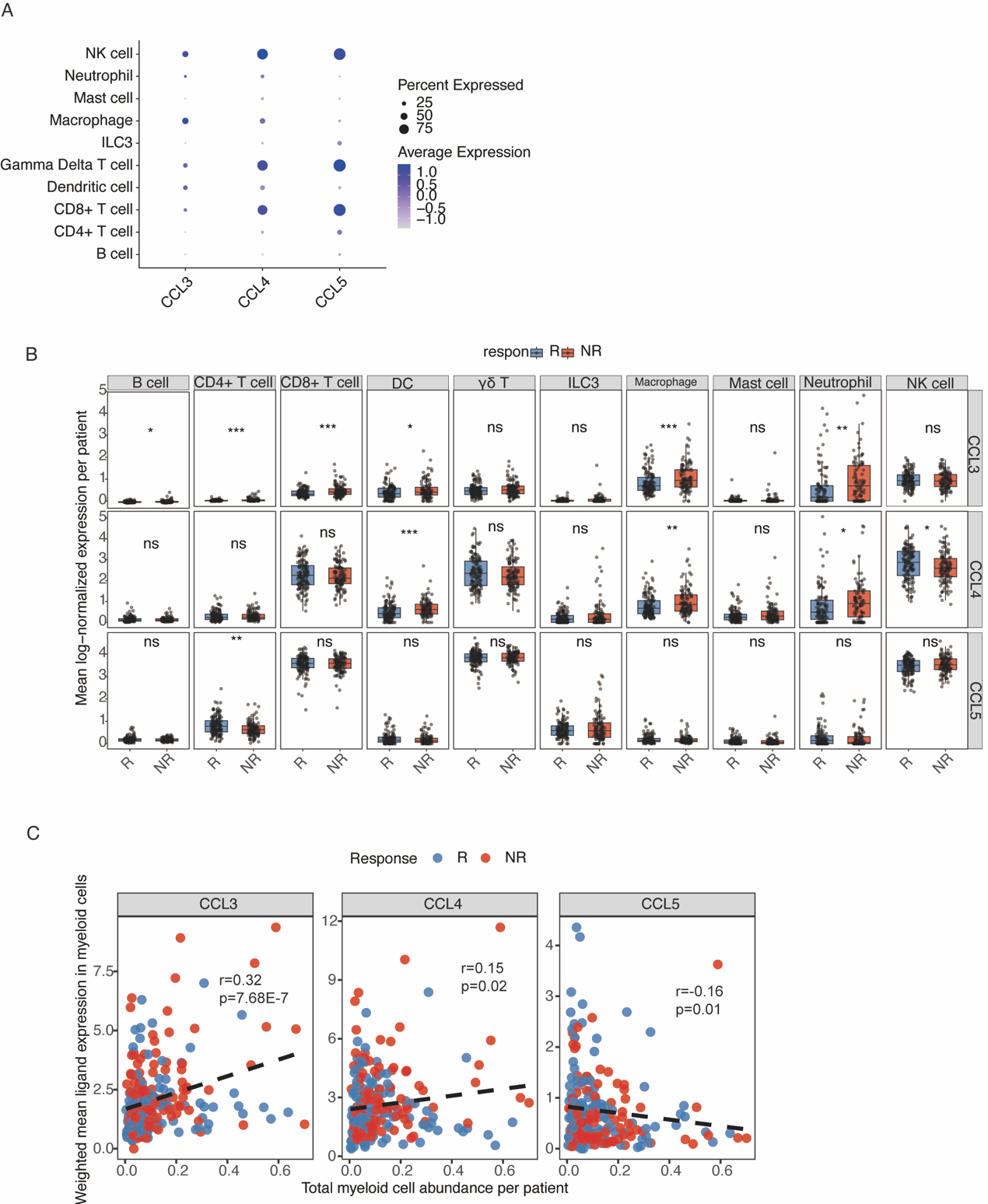
**

**Figure S4. Expression profiles of CCL3, CCL4, and CCL5 and their correlation with clinical response and myeloid abundance.**(A) Dot plot displaying the baseline expression levels and percentages of CCL3, CCL4, and CCL5 across major immune cell types from NSCLC cohort.(B) Stratified box plots showing the mean expression of CCL3, CCL4, and CCL5 per patient across ten distinct cell types, compared between R(N=130) and NR(N=112) samples. Individual dots represent patient samples. (C) Scatter plots illustrating the correlation between total myeloid cell abundance per patient and the weighted mean ligand expression in myeloid cells for CCL3, CCL4, and CCL5.Pearson correlation coefficient (r) and the corresponding P-value are indicated within each panel. Statistical significance was determined by the [Wilcoxon rank-sum test]; ns, not significant; * P < 0.05, **P < 0.02, *** P < 0.001.
